# Music Aptitude, Training, and Cognitive Transfer: A Mini-Review

**DOI:** 10.3389/fpsyg.2022.903920

**Published:** 2022-06-29

**Authors:** Lu Wang

**Affiliations:** Department of Educational Psychology, Ball State University, Muncie, IN, United States

**Keywords:** music aptitude, training, cognitive transfer, neuroplasticity, nature and nurture

## Abstract

In this mini-review, the genetic basis of music aptitude and the effects of music training are discussed. The review indicates that regardless of levels of innate ability, experience-induced neuroplasticity can occur as a result of music training. When that happens, it can be expressed as functional or structural brain changes. These changes are often accompanied by improvement in performance in tasks involving auditory analysis. Specifically, music training effects can transfer to a closely related cognitive domain such as auditory processing (near transfer). Music training can also affect more distantly related cognitive domains such as spatial and linguistic domains. Lastly, music training can affect general intelligence (“g”) (far transfer). Music training can mold behavioral brain development and confers cognitive benefits beyond music.

## Introduction

Depending on one’s theoretical inclination, music aptitude has been defined in terms of collections of interrelated but distinct skills that involve auditory sensory discrimination around pitch, loudness, rhythm, time, timbre, and tonal memory (Seashore, 1938) or as the collective whole and the dynamic interplays of various auditory discrimination and memory skills. In a nutshell, the former camp endorses an atomistic view of music aptitude that compartmentalizes it into six subsets of skills, whereas the latter camp endorses an omnibus view of music aptitude that emphasizes on unity of the skills required to render a beautiful piece of music on a musical instrument (Gordon, 1969). Both camps agree that music aptitude is at least partially genetically determined (nature). There is a difference in view regarding the extent music aptitude can be improved through training (experience-induced neuroplasticity). This mini-review provides evidence that supports either position and evaluates evidence against the notion of whether effects of music training are transferable to closely related (near transfer), somewhat related (medium-distance transfer), and remotely related domains (far transfer).

The mini-review starts by examining the genetic basis of music aptitude. The effects of music training are then discussed in the context of neuroplasticity research using neuroimaging and behavioral studies. Next, findings from training studies that reveal a transfer of music training to closely related and more distantly related cognitive domains are evaluated. These transfer effects illustrate the extent of experience-induced neuroplasticity through music training (nurture).

### Nature and Nurture

There is some evidence that the brain basis of music aptitude is genetically determined (see Richardson, 1990; Freeman, 1999; Gagné, 1999; Strand and Gault, 2014). This genetic evidence highlights nature’s contribution to music aptitude. This evidence includes identified genes that are responsible for auditory discrimination, memory, and the inclination to practice (see Liu et al., 2016; Wesseldijk et al., 2021a). This evidence is reviewed in greater depth in the following section. There is also evidence that highlights nurture’s contribution to music aptitude. This evidence reveals the malleability of music aptitude through training (experience-induced neuroplasticity). Schlaug et al. (2005), for instance, showed trends of functional and structural changes in the brain as soon as 1 year after instrumental training where little preexisting differences in brain activation patterns or structural differences were observed prior to or at the beginning of the music training. In a follow-up fMRI study, Hyde et al. (2009) reported structural brain changes in 6-year-olds who participated in 15 months of music training. These structural brain changes were also accompanied by behavioral improvements in fine motor control and auditory skills. For example, areas of the brain that are responsible for melodic and rhythmic processing showed activation and structural changes after music training. Specifically, areas of the brain that are associated with music perception and cognition showed structural enlargement or changes in activation patterns after music training, rather than predisposing certain individuals to participate in music training. These findings delineate nurture’s contribution to the development of music aptitude.

#### Genetic Basis of Music Aptitude

Researchers who emphasizes on nature’s contribution to music aptitude cited genetic studies to bolster their view. Within this camp, some are convinced that experience-induced neuroplasticity through music training cannot extend inborn music aptitude, whereas others are open to such possibilities. Mosing et al. (2014a) belong to the former camp. These researchers argue that music practice does not causally influence music aptitude. Rather, individual differences in genetics affect both music aptitude and the inclination to practice. In another paper, Mosing et al. (2014b) showed that the genetic basis of music ability included genes that are specifically responsible for auditory functions and genes that also affect general cognitive ability. More recently, Wesseldijk et al. (2021a) identified a partial association between music aptitude and verbal ability after correcting for general intelligence. Shared genetic factors explained 50% of this association and another 35% was explained by non-shared environmental influences. Wesseldijk et al. (2021b) argues that the results from their co-twin-control-analysis suggest music and verbal abilities may share an underlying genetic etiology, rather than music training at a young age causing cognitive transfer in a verbal domain at the age of 18. The research group published another paper (see Wesseldijk et al., 2021a) that discusses the reasons why an early start in music training contribute to musical skills in adulthood. The paper reiterated the genetic contribution of music aptitude, while at the same time placing a greater emphasis on nature and nurture interaction in developing musical skills into adulthood, with an emphasis placed on familial factors. Musically inclined parents not only pass on their musical genes to their offspring but are also more likely to provide family environment that is conducive to developing musical skills and invest in music lessons.

Other recent research on the molecular and evolutionary backgrounds of music aptitude included Liu et al.’s. (2016) work, a case control study that used HploPS, XP-EHH, and FST methods and compared genome-wide genotyping data (641?K SNPs) in 148 Finnish individuals, among which there are 74 cases of high music aptitude individuals. HaploPS is a method of locating genomic evidence by using the phased haplotypes of multiple samples from a population (Liu et al., 2013). The XP-EHH test is a method of detecting “selective sweeps in which the selected allele has risen to high frequency or fixation in one population, but remains polymorphic in the human population as a whole” (Sabeti et al., 2007). FST as a method reflects on the genetic differentiation among populations and shed light on the evolutionary properties of the genetic structure of the populations being studied (Whitlock and McCauley, 1999). Having assigned three positive selection regions, gene ontology classification revealed that the positive selection regions contained genes that affect inner-ear development that are critical to music perception. Liu et al.’s. (2016) literature review also identified adhesion G protein-coupled receptor (*ADGRV1*), also known as G protein-coupled receptor 98 (*GPR98*) and usherin (*USH2A*), as contributing to auditory perception; glutamate ionotropic receptor NMDA type subunit 2B (*GRIN2B*), interleukin 1 alpha (*IL1A*), interleukin 1 beta (*IL1B*), and Rap guanine nucleotide exchange factor 5 (*RAPGEF5*) as contributing to general cognition and memory; forkhead box P1 (*FOXP1*), regulator of G protein signaling 9 (*RGS9*), *GPR98*, and *GRIN2B* as contributing to song perception. Like Oikkonen et al. (2015), Liu et al. (2016) sought for genetic markers of music aptitude. Oikkonen et al. (2015) identified genes affecting inner ear development and brain functions corresponding to music perception. Alpha-synuclein gene (*SNCA*) was found to be overexpressed when listening to and performing music (Kanduri et al. 2015). The GATA-binding protein 2 (*GATA2*) regulates *SNCA* in dopaminergic neurons. *GATA2* links DNA- and RNA-studies of music aptitude [see Oikkonen and Järvelä (2014) review for related genetic studies of music aptitude; see also Tan et al. (2014) for a comprehensive review on behavioral and molecular genetic studies of music aptitude]. Genomic analyses conducted by Park et al. (2012) research group on 1008 Mongolian individuals identified an intergenic single nucleotide polymorphism (SNP) and a non-synonymous SNP in UDP Glycosyltransferase 8 (*UGT8*) to be strong determinants of music aptitude, as assessed by a pitch-production accuracy test.

#### Near Transfer, Medium-Distance Transfer, and Far Transfer

A trait’s having a genetic basis does not preclude experience-induced neuroplasticity from taking place. In other words, if a child is genetically endowed with high music aptitude, it does not preclude the possibility that through quality instruction and deliberate practice, the child can extend that innate aptitude further. A body of research supports experience-induced neuroplasticity, both at the functional/structural brain level and at the behavioral level. This section discusses findings concerning “near transfer,” a transfer of music training to closely related cognitive domains, “medium-distance transfer,” a transfer of music training to somewhat related cognitive domains, and “far transfer,” a domain-general transfer of music training. In this discussion, music experience is limited to active music experience. This means “the Mozart effect,” which results from music listening, a passive music experience, is beyond the scope of this manuscript.

The Mozart effect refers to a temporal boost of spatial-temporal reasoning after being exposed to a Mozart sonata K448 for 10 min (see Rauscher et al., 1993). This effect spurred a craze about listening to Mozart, classical music, or compositions that show regularities similar to that of Mozart sonata K448. The effect was replicated by a few independent research labs, though even in studies where positive findings were reported, the effect size was small and short-lived (lasts about 12 min). Also, the effect size varies depending on the test that was used to measure spatial ability (Jenkins, 2001). Furthermore, for every successful replication of the Mozart effect, there are about just as many studies that failed to reproduce the Mozart effect. The Mozart effect is considered a passive listening effect. Unlike the Mozart effect, however, the music training effect concerns active participation in music, e.g., receiving music instruction, engaging in deliberate practice.

##### Near Transfer (Auditory Processing Skills)

Music training, especially when having an early start, was intensive, or lasted for some time, confers cognitive, emotional, and physiological benefits. This manuscript focuses on the effects of music training on cognition. Improvement in auditory processing skills exemplifies near transfer, improvement in verbal and visuospatial abilities illustrates medium-distance transfer, and enhanced executive functions and “g” instantiates the occurrence of far transfer.

Tervaniemi et al. (2011) identified the neural signatures (MMNm) of long-term music training (musical expertise) using magnetoencephalographic (MEG). These neural signatures included the left auditory areas. The researchers also found that musically trained and musically talented groups engage both auditory cortices in an integrative manner. Tervaniemi et al. (2011) study showed that music training and music aptitude have similar neural signatures during chord discrimination. However, their findings cannot answer the question of whether structural and activation differences in the brain is due to postnatal training or innate aptitude.

Direct evidence supporting near transfer is provided by Kuchenbuch et al. (2012) study. Also using magnetoencephalography (MEG), the research group investigated the influence of long-term music training on the processing of partly imagined tone patterns (imagery condition) and identical but perceived patterns (perceptual condition). The magnetic counterpart of mismatch negativity (MMNm) of musicians and non-musicians were recorded as they performed a lab devised task requiring them to detect deviant tone patterns. The results showed that the latency and the laterality of the MMNm in the perceptual condition differed significantly between the groups, with musicians having an earlier MMNm onset, especially in the left hemisphere. At a behavioral level, the musician group outperformed the non-musician group in detecting deviant tones (perceptual condition) and imaging deviant tones (imagery condition). Kuchenbuch et al. (2012) concluded that processing patterns is faster and more strongly lateralized in musicians. These findings lent direct support for near transfer as a result of long-term music training.

Banai and Ahissar (2013) proposes that the extent music training transfer into other cognitive domains and the degree of generalization may be subjective to a threshold effect. Individuals with poor memory and auditory discrimination skills are likely to experience a broader generalization or cross-domain transfer as a result of music training than their high ability counterpart. Also, they believe that these individuals’ auditory and memory skills are also likely to show a more pronounced positive association with their music-related skills. Due to a correlational design, however, Banai and Ahissar (2013) is unable to definitively conclude a causal effect from music training to auditory processing and, subsequently, verbal skills such as reading.

##### Medium-Distance Transfer (Verbal and Visuospatial Domains)

In between studies illustrating direct transfer (near transfer) and domain-general transfer (far transfer) are cross-domain transfer (medium-distance transfer). In this section, studies supporting cross-domain transfers from music to verbal and visuospatial domains are discussed. Not only does music and language share an evolutionary history, music also has a special affinity with the phonetic aspect of language. Mackenzie Beck (2003) and Dankovičová et al. (2007), for instance, showed that music aptitude is predictive of phonetic skills. Phonetic skills are a modular aspect of language.

Articulatory rehearsal is another modular aspect of language. Degé and Schwarzer (2017) compared musically trained 10–12-year-old children on two verbal working memory tests, one involving memorizing a word list under a normal condition and the other involving memorizing a similar word list under an articulatory suppression condition. After controlling for gender, socioeconomic status (SES), general intelligence, motivation, music aptitude, and personality, musically trained children still outperformed untrained children under normal but not under articulatory suppression condition, suggesting that music training enhances linguistic ability through improving articulatory rehearsal. Articulatory rehearsal is involved during short-term storage of word lists.

Forgeard et al. (2008) study not only provided evidence for near transfer from music to motor skills and auditory processing, but also cross-domain (medium-distance) transfer. Beside auditory discrimination and motor skills tests, children who participated in the study also completed batteries of non-verbal reasoning (Raven’s Progressive Matrices), a verbal ability test (Vocabulary subtest from the WISC-III), two spatial ability tests (Block Design subtest of the WISC-III and Object Assembly subtest of the WISC-III), and a mathematics achievement test (Keymath-Revised: A Diagnostic Inventory of Essential Mathematics). The results of the analyses showed that musically trained group outperformed their untrained counterpart in verbal and non-verbal reasoning. It should be noted that unlike Rauscher et al. (1997) study, discussed in greater detail in the ensuing paragraph, Forgeard et al. (2008) study did not find music training to improve spatial ability. Nor was there strong evidence for improvement in mathematics achievement in musically trained children.

Although from Forgeard et al. (2008) study, it appears as though the effects of music training has a limited transferability to other cognitive domains (in this study, it is verbal and non-verbal reasoning, but not spatial and mathematics), and the fact that the study was correlational by design and hence cannot be used as evidence for a causal relationship, Rauscher et al. (1997) study, discussed in greater detail in the ensuing paragraph, is experimental by design and showed a causal relationship between music training and enhanced spatial-temporal reasoning in young children. Similarly, a few other studies are suggestive of a positive association between music training and enhanced math-related skills as well (see Cheek and Smith, 1999; Ribeiro and Santos, 2017; Rodriguez et al., 2019). Cheek and Smith (1999), although correlational by design, showed a positive association between 2 years of private music lessons and enhanced numerical skills in typically developing adolescents. Ribeiro and Santos (2017) studies, although quasi-experimental by design, also showed positive associations between music training and subsets of numerical skills. Specifically, both typically developing (TD) and the group with developmental dyscalculia (DD), a condition marked by impoverished numerical skills, performed significantly better on number comprehension, number production, and calculation tests after a 7-lesson music training program that targeted core skills in music such as rhythms, melodies, and harmonies perception than their baseline performance on the same numerical tests prior to the music training.

The most compelling evidence for the cross-domain (Medium-Distance) transfer come from two studies that used a variant of the experimental design. Using a double-blind prospective case-control study, Rodriguez et al. (2019) studied the effects of eight group sessions of Numeracy Music training (NMT) on 42 school children (age ranged from 8 to 10)’s numerical cognition, working memory, and math anxiety. The results showed a reduction in math anxiety, as well as significant improvements in performance on numerical cognition and working memory tests after the training. Another experimental study that enables a causal inference of transfer of music training to another cognitive domain (spatial cognition) to be drawn comes from Rauscher et al. (1997) study. Rauscher et al. (1997) investigated the impact of music training on spatial recognition and spatial-temporal reasoning in preschool children who enrolled in keyboard lessons. Rauscher et al. (1997) were among the first to provide experimental evidence for a cross-domain transfer from music training to certain spatial skills. The study showed that music training, even of a short duration, improves spatial-temporal reasoning (aka mental rotation, spatial imagery) but not spatial recognition (aka object recognition, visual ability). The magnitude of improvement in spatial-temporal reasoning was greater than one standard deviation. The effect lasted for more than 1 day.

The discrepancy between Rauscher et al. (1997) and Forgeard et al. (2008) findings concerning the effect of music training on spatial temporal reasoning may be due to musically trained individuals have improved their spatial ability initially. However, at some point, the developmental advantage of music training may taper off. The non-musically trained group catches up. The null finding reported by Forgeard et al. (2008) may also be due to the Block Design subtest not being sensitive enough to pick up the training effects. This is because in other studies that used different instruments to measure spatial-temporal reasoning (see Rauscher et al., 1997; Sarnthein et al., 1997), musically trained individuals showed better spatial-temporal reasoning.

In a causal-comparative (a quasi-experimental design) study, Wang et al. (2018) found a positive association between music training and perspective-taking and spatial imagery abilities in young adults attending 4-year colleges. The Perspective-Taking Ability (PTA) test measures the ability to navigate in real or virtual large-scale space. The Spatial Imagery Ability (SIA) test measures the ability to mentally represent, temporarily store, and manipulate visuospatial information along an allocentric frame of reference.

##### Far Transfer (Executive Functions and General Intelligence/“g” Factor)

There is also some evidence for far transfer from music to domain-general cognition (Brandler and Rammsayer, 2003; Helmbold et al., 2005). Music training is positively associated with executive functions and “g” (Degé et al., 2011; Okada and Slevc, 2016). Childhood music training predicts better mathematics, language, and spatial skills (see Schellenberg, 2005; Rauscher and Hinton, 2011; Schellenberg and Winner, 2011). However, there are also studies that failed to find positive music training effects on domain-general cognition (see Schellenberg, 2011).

In a selective review of neuroimaging studies that investigated the neural signatures of cognitive training, Putkinen and Saarikivi (2018) found decreases in frontoparietal activities after music training. The researchers concluded that musically trained individuals have more mature executive functions that are characterized by more efficient information processing in frontoparietal areas. This greater efficiency in information processing corresponds to less activation in frontoparietal network, which undergirds executive functions. The researchers then presented their preliminary findings of an fMRI study that explored executive functions development. Consistent with their prediction, musically trained adolescents and young adults showed fewer frontoparietal brain activations than their untrained counterparts when performing challenging executive functions tasks. This neural signature was accompanied by a behavioral advantage in the musically trained group. In the same study, Putkinen and Saarikivi (2018) also measured general intelligence, using a combination of verbal and visuospatial tests, but did not find group differences.

In light of the mixed findings concerning music training and general intelligence, Schellenberg and Moreno (2010) conducted a study using a full-scale Advanced Raven’s Progressive Matrices to measure “g.” Raven’s Progressive Matrices and Advanced Raven’s Progressive Matrices are considered a gold standard to capture “g.” Their results failed to reveal significant group differences in “g” between musically trained and untrained individuals.

Hargreaves and Aksentijevic (2011) and Okada and Slevc (2016) discussed factors that could contribute to mixed findings on far transfer. Some of these factors are: (i) the correlations between music training and IQ may vary at different levels of music aptitude; (ii) socio-economic status (SES) differences between musically trained and untrained group, though sometimes statistically controlled for, may still influence the results of the studies; (iii) differences in parental educational background between musically trained and untrained group are often unaccounted for; (iv) the effects of music training on general intelligence may differ across age groups; (v) the effects of music training on general intelligence may vary among individuals with different neurological conditions.

Perhaps most importantly, controversies concerning the effect of music training on general intelligence stems from the age-old “chicken-and-egg” problem. Group differences in general intelligence could be due to preexisting differences in intellectual profiles. Individuals who are drawn to music may have higher IQs to start with (see Swaminathan et al., 2017), rather than music training improving general intelligence (Schellenberg and Moreno, 2010; Schellenberg, 2011). To effectively argue that the direction of effect is from music training to general intelligence, experimental, longitudinal, and neuroimaging studies are needed.

#### Experience-Induced Neuroplasticity Through Music Training and the Role of a Sensitive Period

In this section, how neuroplasticity occurs as a result of music training and its possible interaction with a sensitive period is explained. Prior research showed that bilingualism, participation in some sports activities, and even experience working as a taxi driver in London for a certain amount of time can induce functional or structural changes in the brain (see Maguire et al., 2000; Habibi et al., 2018). Recent research in the area of language development (see Turker et al., 2021) suggests that a sensitive period exists in language learning. Inter-individual differences in language learning outcomes over the course of development may be explained in terms of the interaction between neuroplasticity and sensitive period. Certain aspects of language (e.g., the phonetic aspect of second language acquisition) may be constrained by a sensitive period. In other words, language acquisition, whether L1 or L2, is more efficient during the sensitive period, although individual differences in linguistic aptitude and motivational factors also play important roles. In Turker et al.’s (2021) words, the interaction between experiential and maturational factors could impact language learning across the lifespan. Late L2 acquisition is constrained on the neural level by “maturational declines in synaptic density, decreased levels of brain metabolism (Bates et al., 1992), and increased axon myelination (Pulvermüller and Schumann, 1994).”

Although music is not singular in inducing neuroplasticity, it is unique in its complexity and the extent it engages the whole brain (Herholz and Zatorre, 2012). For that reason, some suggest that professional musicians’ brains are a good model to study lifespan neuroplasticity (Münte et al., 2002; Rodrigues et al., 2010; Herholz and Zatorre, 2012). Studies published thus far explored neuroplasticity as a result of music training using a variety of structural and functional neuroimaging techniques including MRI, EEG, fMRI, PET, and DTI (Münte et al., 2002). Some of these studies showed changes in brain activation patterns after just a short period of training (see Rauscher et al., 1997; Rauscher and Zupan, 2000; Norton et al., 2005; Hyde et al., 2009). There is also evidence that intensive long-term music training can induce morphological changes in gray matters from this literature (see Herholz and Zatorre, 2012). What remains to be explored further, however, is the precise time window, the domain-specificity of sensitive period, and the neural instantiations of the interaction between neuroplasticity and sensitive period concerning music training. Given that some aspects of language learning (e.g., phonetics) resembles music learning (e.g., pitch identification or discrimination) and engages the same brain regions, there is a good reason to speculate that the sensitive period for music training at least partially overlaps with that of language acquisition.

In sum, key findings and directions for further research on the topic of experience-induced neuroplasticity and the role of sensitive period in music training are: (i) structural brain changes in adult professional instrumentalists can be predicted by intensity of training, starting age of training, and duration of training (Merrett and Wilson, 2012); (ii) there are large inter-individual variabilities in response to music training; (iii) the effects of music training may vary by types of music training (e.g., instruments, instructional formats, see Reybrouck and Brattico, 2015); (iv) the direction of causal effects between music training and general intelligence remains to be experimentally determined (Schellenberg and Peretz, 2007); (v) due to similarities between some aspects of language acquisition and music learning, music training’s effectiveness will likely be subject to the influence of sensitive period—a time window that demarcates a high level of receptivity and efficiency in learning. The effects of music training will likely to be more pronounced when music training takes place during the sensitive period (White et al., 2013).

## Conclusion

This mini-review provides a bird eye view of the interdisciplinary field of mind, brain, and education on music aptitude, training, and cognitive transfer. Insights gained from synthesizing these bodies of works can inform educational neuroscience research concerning how gene-environmental interactions contribute to the unfolding of music aptitude across the lifespan, with sensitive period moderating the malleability of music aptitude through training and domain similarity predicting the magnitude of cognitive transfer. Overall, this mini review indicates that regardless of levels of innate aptitude, experience-induced neuroplasticity can occur as a result of music training and may be subject to the influence of sensitive period. Experience-induced neuroplasticity can be expressed as functional or structural brain changes (e.g., Schneider et al., 2002; Schlaug et al., 2005) that are often accompanied by enhanced behavioral performance (e.g., Hyde et al., 2009). The right auditory cortex, for example, has been shown to be malleable to music training (see Schneider et al., 2002) and is a good candidate for further investigations and replications.

Music training can directly transfer to closely related cognitive domains such as auditory processing (near transfer). Training effects can also leak into more distantly related cognitive domains such as certain aspects of spatial and linguistic skills *via* medium-distance transfer. More experimental and longitudinal research is needed to establish the neural basis, behavioral manifestations, and causal mechanisms of medium-distance transfer from music to related cognitive domains that include language, mathematics, and spatial reasoning. Finally, music training can also affect general intelligence *via* far transfer.

Neuroplasticity research generally supports the notion that music training, especially when started early, was intensive, and lasted for several years, leave distinct neural signatures in the brain, accompanied by behavioral performance differences between musically trained and untrained groups (Rodrigues et al., 2010). Playing an instrument engages the whole brain that includes auditory processing, motor controls, executive functions, and bi-hemispheric communications. It therefore provides an ideal model to study how nature (music aptitude) and nurture (music training) work in tandem (White et al., 2013) in the making of a musician.

The nature of sensitive period as expressed in music learning and how neuroplasticity moderates the training effects on the expression of music aptitude (as outlined in the previous section) warrant further research using experimental, longitudinal, and neuroimaging methods. This research will also refine our understanding of issues with broader implications such as mind-brain relationship, the architecture of the mind, and the causal mechanism of the transfer of training effects at the neuronal and behavioral levels. This mini-review suggests that music training molds behavioral brain development, regardless of innate levels of music aptitude, and confers cognitive benefits beyond music. The logical next-step in this research is to pinpoint the time window during which cognitive transfer effects are most robust.

Lastly, it should be noted that not all studies cited in support of some of the arguments made are independent from one another, meaning some studies cited are follow-up from a previous study and was led by a different researcher within the same research group (e.g., studies conducted by Järvelä and colleagues and Ullen and colleagues). Meta-analysts are advised to use cautions when polling studies directly from this mini-review’s reference list without discerning the possible presence of dependency in findings reported by different authors within the same research group.

## Author Contributions

The author confirms being the sole contributor of this work and has approved it for publication.

## Conflict of Interest

The author declares that the research was conducted in the absence of any commercial or financial relationships that could be construed as a potential conflict of interest.

## Publisher’s Note

All claims expressed in this article are solely those of the authors and do not necessarily represent those of their affiliated organizations, or those of the publisher, the editors and the reviewers. Any product that may be evaluated in this article, or claim that may be made by its manufacturer, is not guaranteed or endorsed by the publisher.
